# Assessment of Surgeon Factors Associated With Margin Re-excision After Breast-Conserving Surgery

**DOI:** 10.1001/jamanetworkopen.2022.28100

**Published:** 2022-08-22

**Authors:** Jeffery M. Chakedis, Sharon B. Chang, Annie Tang, Gillian E. Kuehner, Alison C. Savitz, Brooke Vuong, Maihgan A. Kavanagh

**Affiliations:** 1 Department of General Surgery, The Permanente Medical Group, Oakland, California; 2 Department of Surgery, University of California San Francisco, East Bay, Oakland

## Abstract

This cross-sectional study examines 5-year re-excision rates and use of recommended techniques among breast surgeons in a single health system.

## Introduction

Re-excision after breast-conserving surgery (BCS) is a common problem, and high rates of reoperation are concerning because they are associated with adverse outcomes and treatment experiences.^1,2^ To address this problem, the American Society of Breast Surgeons (ASBrS) published techniques surgeons can use to reduce re-excision rates.^3^ These techniques include localization for nonpalpable tumors, multidisciplinary review, specimen orientation and intraoperative radiography, oncoplastic techniques, cavity shave margins, intraoperative pathologic review, and margin guidelines compliance. At Kaiser Permanente Northern California, we evaluated the association between surgeon practices and rates of re-excision after BCS to identify successful techniques.

## Methods

Patients with newly diagnosed primary breast cancer who underwent BCS between January 1, 2016, and December 31, 2020, were identified from a prospectively collected registry. Institutional review board approval and the informed consent requirement were waived by the Research Determination Committee for Kaiser Permanente Northern California. We followed the STROBE reporting guideline for cross-sectional studies.

Patient-level data were obtained from electronic medical records. Surgeon demographic characteristics, practice volume and composition, and ASBrS technique utilization data were obtained by surveying current breast surgeons. Univariable and multivariable linear regression analyses were performed to identify factors associated with decreased re-excision rates. A 2-sided *P* = .05 was indicated significance. 

## Results

Over a 5-year period, 55 surgeons (18 men [33%] and 37 women [67%]) performed 9054 BCS with a re-excision rate of 18.8%. Individual surgeon’s 5-year re-excision rates ranged from 7.8% to 36.8%, with a 3.3-fold difference between the 10th and 90th percentile (Figure). In the survey, 53% of surgeons reported being in practice for more than 10 years, 31% having fellowship training (breast or surgical oncology), and 45% having breast-focused (>50% of cases) specialty practices.

**Figure.**
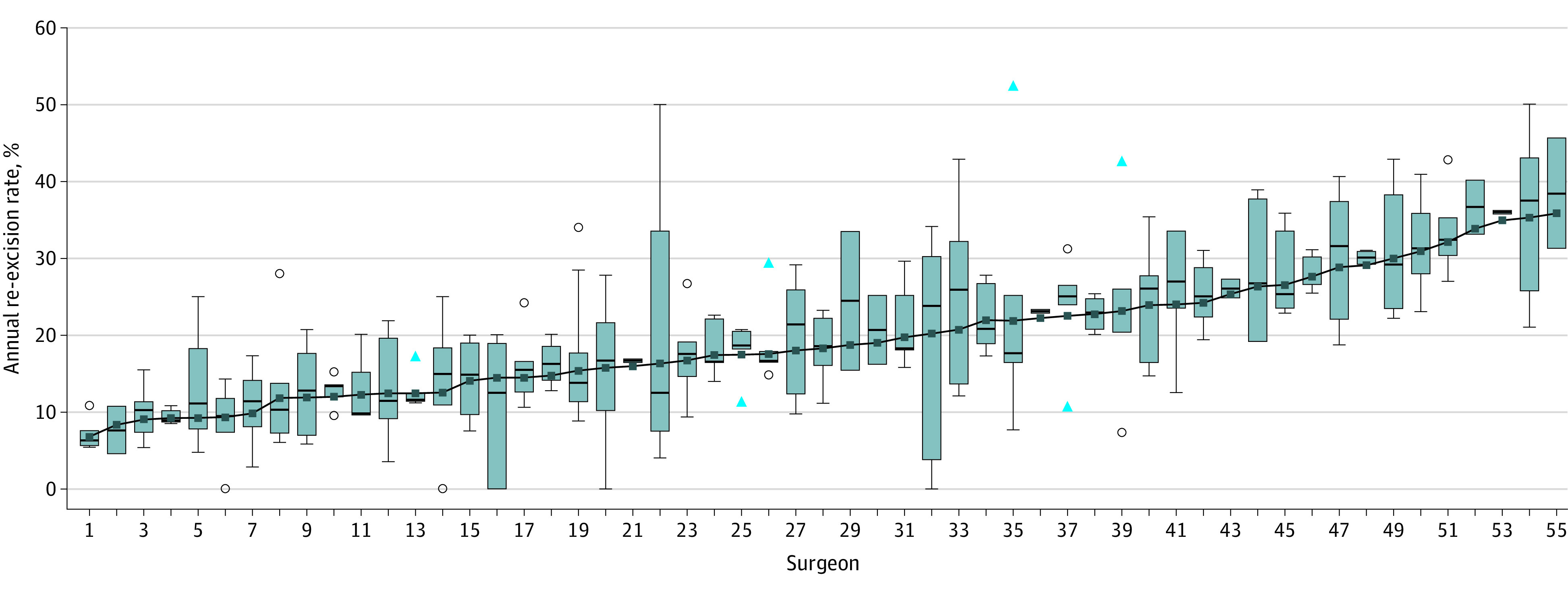
Five-Year Re-excision Rates for Individual Surgeons The solid marker and trend line represent the mean rate, box and whisker plot represent the median rate with IQR, and circles and triangles represent outlier values of the median.

As shown in the Table, surgeon factors associated with lower re-excision rates included some ASBrS techniques (Society of Surgical Oncology and American Society for Radiation Oncology guidelines, routine use of oncoplastic techniques, intraoperative margin analysis, and always use of cavity shave margins), increased operative volume, and neoadjuvant chemotherapy. We compared surgeons who always used shave margins with those who did not (10 vs 45) and found no differences in their practice patterns or operative volumes. Surgeons who always used cavity shave margins had lower mean re-excision rates (14.1% vs 21.7%; *P* = .004). Since the onset of cavity shave margin use in a subset of patients in 2018 (n = 4803), shave margins have been used in 18% of patients. Re-excision rate was lower in BCS in which shave margins were used compared with BCS without shave margins (13.9% vs 19.4%; *P* < .001).

**Table.** Factors Associated With Lower Re-excision Rates in Univariable and Multivariable Linear Regression Models

Surgeon-specific variable	Univariable model		Multivariable model	
	β (95% CI)	*P* value	β (95% CI)	*P* value
Mastectomy rate	0.22 (−0.06 to 0.51)	.12	0.14 (−0.13 to 0.41)	.29
Percentage of operations for DCIS	0.18 (−0.25 to 0.61)	.42	0.13 (−0.25 to 0.51)	.49
SSO-ASTRO margin guidelines	−1.95 (−8.44 to 4.55)	.55	−2.05 (−7.58 to 3.48)	.46
BCS per year	−0.11 (−0.21 to −0.006)	.04	−0.04 (−0.15 to 0.06)	.42
Neoadjuvant chemotherapy use	−0.62 (−1.09 to −0.16)	.01	−0.48 (−0.94 to −0.02)	.04
Ultrasonography-guided localization use	−5.64 (−10.2 to −1.11)	.02	−2.89 (−7.06 to 1.28)	.17
Medical center location	0.47 (0.02 to 0.92)	.04	0.34 (−0.9 to 0.76)	.12
Breast-focused practice (>50% of total cases)	−4.46 (−8.65 to −0.27)	.04	−0.06 (−4.86 to 4.74)	.98
Routine or always use of oncoplastic techniques	−6.25 (−10.5 to −2.01)	.005	−2.05 (−6.19 to 2.09)	.32
Intraoperative gross margin analysis	−6.19 (−11.6 to −0.81)	.03	−4.86 (−9.86 to 0.13)	.06
Always use of cavity shave margins	−7.65 (−12.9 to −2.42)	.005	−6.11 (−11.0 to −1.64)	.009

Abbreviations: BCS, breast-conserving surgery; DCIS, ductal carcinoma in situ; SSO-ASTRO, Society of Surgical Oncology and American Society for Radiation Oncology.

Patient characteristics in the shave vs no-shave margin cohorts were similar and differed only in the percentage with breast cancer history (4.9% vs 7.6%; *P* = .004) and neoadjuvant chemotherapy use (7.8% vs 5.5%; *P* = .01). In the multivariable model adjusted for patient and surgeon factors, always use of shave margins was an independent factor in lower re-excision rates (β, −6.11; 95% CI, −11.0 to −1.64; *P* = .02).

## Discussion

We found significant variability in re-excision rates and surgical practices among breast surgeons, which are consistent with US averages.^1^ Patient, surgeon, and facility factors were all associated with the need for re-excision, and age, breast density, tumor size, and ductal carcinoma in situ or lobular histologic feature were previously identified as patient factors in this cohort (J.M. Chakedis, MD, unpublished data, 2022). Results align with those of 2 randomized clinical trials, which showed routine use of shave margins decreased re-excision rates by 50%.^4,5^ The limitations of the present study include re-excision use as a correlate for involved pathologic margins.

We identified surgeon factors that were associated with lower re-excision rates and cavity shave margins as the most effective technique. We provided practical evidence of the benefits of routine cavity shave margins, which is now a standard practice among breast surgeons at our health system.
